# Fault detection of gearbox by multivariate extended variational mode decomposition-based time–frequency images and incremental RVM algorithm

**DOI:** 10.1038/s41598-023-34868-4

**Published:** 2023-05-16

**Authors:** Siwei Nao, Yan Wang

**Affiliations:** 1 Medical Technology School, Qiqihar Medical University, Qiqihar, 161000 China; 2 Basic Medical Science School, Qiqihar Medical University, Qiqihar, 161000 China

**Keywords:** Engineering, Mathematics and computing

## Abstract

A novel detection method based on multivariate extended variational mode decomposition-based time–frequency images and incremental RVM algorithm (MEVMDTFI–IRVM) is presented for fault detection of gearbox. The time–frequency images are constructed by multivariate extended variational mode decomposition. Compared with single-variable modal decomposition method, multivariate extended variational mode decomposition not only has an accurate mathematical framework, but also has good robustness to non-stationary multi-channel signals with low signal-to-noise ratio. The incremental RVM algorithm is presented for fault detection of gearbox based on the time–frequency images constructed by multivariate extended variational mode decomposition. The testing results demonstrate that the detection results of MEVMDTFI–IRVM for gearbox are stable, in addition, the detection results of MEVMDTFI–IRVM for gearbox are better than those of variational mode decomposition-based time–frequency images and incremental RVM algorithm (VMDTFI–IRVM), variational mode decomposition–RVM algorithm (VMD–RVM), and traditional RVM algorithm.

## Introduction

Industrial gear boxes, such as spur gear boxes, spiral gear boxes, bevel gear boxes and planetary gear boxes, are widely used in rotating machinery due to their versatility in power and motion transmission^1–3^. Almost every industry uses these gearboxes to manage various industrial and precise functions^4,5^. However, due to repeated loading and operation under different fault types, such as abrasion, pitting and snaggletooth, will occur in the machine components inside the transmission. These defects worsen over time, leading to abnormal behavior in the vibration signal^6,7^. Feature extraction in the fault diagnosis fields is the key to obtaining high diagnosis results.

Wavelet analysis and empirical mode decomposition are widely applied to feature extraction in the fault diagnosis fields^8,9^. However, there are mode mixing in the wavelet analysis and empirical mode decomposition. Variational mode decomposition can separate harmonic signals close to the frequency range without being affected by the sampling frequency, which can avoid mode mixing^10–12^. Variational mode decomposition is widely applied to feature extraction in the fault diagnosis fields. For instance, Sharma and Parey proposed a feature extraction method of weak fault transients using variational mode decomposition for fault diagnosis of gearbox under varying speed^13^. However, the traditional variational mode decomposition is a kind of single-variable modal decomposition method. Compared with single-variable modal decomposition method, multivariate extended variational mode decomposition not only has an accurate mathematical framework, but also has good robustness to non-stationary multi-channel signals with low signal-to-noise ratio. In order to highlight more features, time–frequency images are used, in this paper, the time–frequency images are constructed by multivariate extended variational mode decomposition.

Relevance vector machine has a better generalization ability than support vector machine due to the less support vectors of RVM than those of SVM, and less training parameters need to be determined^14,15^. In order to improve the generalization ability of relevance vector machine,an incremental relevance vector machine algorithm is presented in this paper. In incremental relevance vector machine algorithm,the original formulation of RVM is extended to design incremental RVM. Incremental learning helps to simplify time calculation and increase algorithm generalization.

Therefore, a novel detection method based on multivariate extended variational mode decomposition-based time–frequency images and incremental RVM algorithm is presented for fault detection of gearbox. The time–frequency images are constructed by multivariate extended variational mode decomposition, and incremental RVM algorithm is used for fault detection of gearbox based on the time–frequency images constructed by multivariate extended variational mode decomposition. The vibration signals of gearbox with healthy, abrasion, pitting and snaggletooth are respectively collected under the situation of small load and big load, respectively. The testing results demonstrate that the detection results of MEVMDTFI–IRVM for gearbox are stable, in addition, the detection results of MEVMDTFI–IRVM for gearbox are better than those of VMDTFI–IRVM, VMD–RVM, and traditional RVM algorithm.

Firstly, the multivariate extended variational mode decomposition method is introduced. Secondly, incremental RVM algorithm is introduced.Thirdly,fault detection process of gearbox by multivariate extended variational mode decomposition-based time–frequency images and incremental RVM algorithm is introduced. Finally,experimental testing and results are introduced, and conclusion is introduced.

## Multivariate extended variational mode decomposition

Variational mode decomposition can separate harmonic signals close to the frequency range without being affected by the sampling frequency, which can avoid mode mixing. Variational mode decomposition is a generalization of Wiener filter in multiple adaptive frequency bands^16,17^. Variational mode decomposition decomposes the original signal into a set of variational mode decomposition signals called VMFs by the follows:

1 $$\mathop {\min }\limits_{{\{ u_{b} \} ,\{ \omega_{b} \} }} \left\{ {\sum\limits_{b} {\left\| {\partial_{t} \left[ {\left( {\theta (t) + \frac{j}{\pi t}} \right) * u_{b} \left( t \right)} \right]e^{{ - j\omega_{b} t}} } \right\|_{2}^{2} } } \right\}$$

subject to the conditions,

$$g\left( t \right) = \sum\limits_{b} {u_{b} \left( t \right)}$$

where * is the convolution operator,$\partial_{t}$ is the time-related partial derivative, and $\theta (t)$ is the Dirac distribution.

Use the quadratic penalty factor and exponential Lagrange multiplier to change the constrained change problem into an unconstrained change problem given by the follows:

2 $$Q\left( {\left\{ {u_{b} } \right\},\left\{ {\omega_{b} } \right\},\psi } \right) = \alpha \sum\limits_{b} {\left\| {\partial_{t} \left[ {\left( {\theta (t) + \frac{j}{\pi t}} \right) * u_{b} \left( t \right)} \right]e^{{ - j\omega_{b} t}} } \right\|_{2}^{2} } + \left\| {g\left( t \right) - \sum\limits_{b} {u_{b} \left( t \right)} } \right\|_{2}^{2} + \left\langle {\psi \left( t \right),g\left( t \right) - \sum\limits_{b} {u_{b} \left( t \right)} } \right\rangle$$

where $\alpha$ is the constraint factor, and $\psi \left( t \right)$ is the Lagrangian multiplier.

Compared with single-variable modal decomposition method, multivariate extended variational mode decomposition has an accurate mathematical framework and good robustness to non-stationary multi-channel signals with low signal-to-noise ratio.

The constrained optimization problem of multivariate extended variational mode decomposition is defined as,

3 $$\mathop {\min }\limits_{{\{ u_{b,c} \} ,\{ \omega_{b} \} }} \left\{ {\sum\limits_{b} {\sum\limits_{c} {\partial_{t} \left\| {u_{b,c} (t)e^{{ - j\omega_{b} t}} } \right\|_{2}^{2} } } } \right\}$$

subject to the conditions,

$$g_{c} \left( t \right) = \sum\limits_{b} {u_{b,c} (t)}$$

where $u_{b,c} (t)$ is the analytical representation of each element in the corresponding channel *c* and $u_{b} (t)$.

Create the Lagrangian representation for the variational problem, which is shown as follows:

4 $$Q\left( {\left\{ {u_{b} } \right\},\left\{ {\omega_{b} } \right\},\psi } \right) = \alpha \sum\limits_{b} {\sum\limits_{c} {\partial_{t} \left\| {u_{b,c} (t)e^{{ - j\omega_{b} t}} } \right\|_{2}^{2} } } + \left\| {g_{c} \left( t \right) - \sum\limits_{b} {u_{b,c} (t)} } \right\|_{2}^{2} + \sum\limits_{c} {\left\langle {\psi \left( t \right),g_{c} \left( t \right) - \sum\limits_{b} {u_{b,c} (t)} } \right\rangle }$$

**Figure 1:**
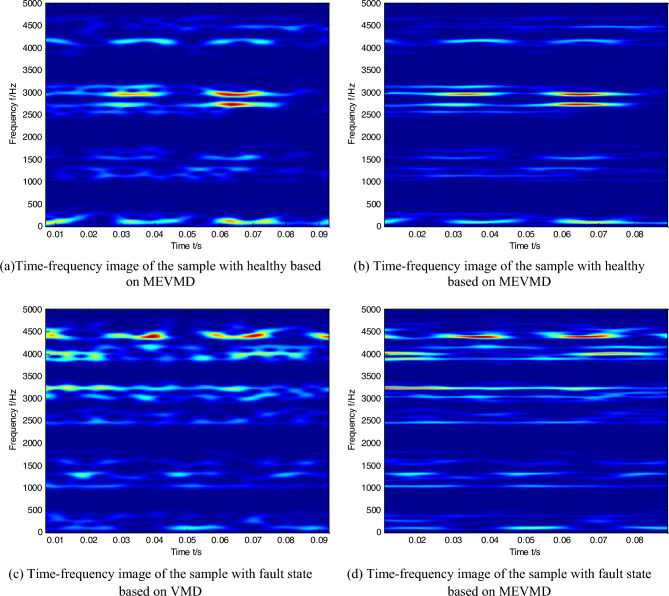
Comparison of time–frequency images between VMD and MEVMD under the situation of small load.

**Figure 2:**
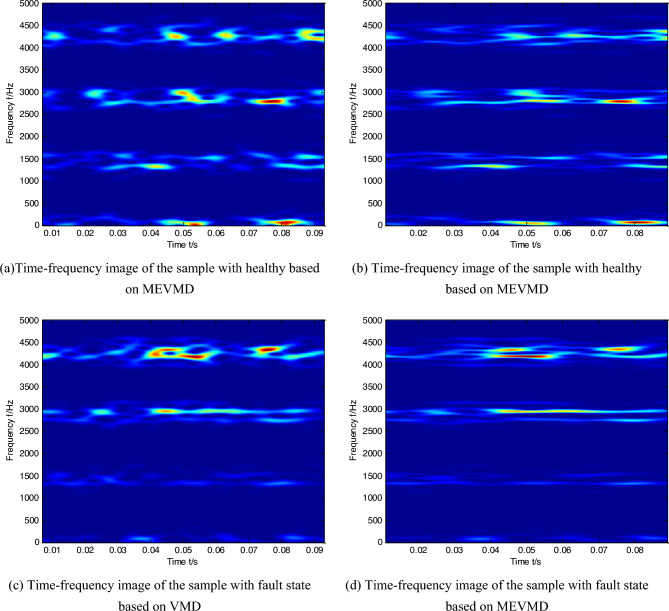
Comparison of time–frequency images between VMD and MEVMD under the situation of big load.

In order to highlight more features, the time–frequency images are structured by multivariate extended variational mode decomposition. The comparison of time–frequency images between VMD and MEVMD under the situation of small load is given Fig. 1, the comparison of time–frequency images between VMD and MEVMD under the situation of big load is given Fig. 2, the abscissa represents time, and the ordinate represents frequency. It can be seen that the features of time–frequency images based MEVMD are clearer than those based on VMD regardless of small load or high load.

## Incremental RVM algorithm

Relevance vector machine has a better generalization ability than support vector machine due to the less support vectors of RVM than those of SVM, and less training parameters need to be determined. Given a set of training samples $\{ x_{i} ,t_{i} \}_{i = 1}^{N}$, where $x_{i}$ presents the input vector, $t_{i} \in \left\{ {0,1} \right\}$. The RVM classification function is defined as follows^18,19^:

5 $$g(x) = \sum\limits_{i = 1}^{N} {w_{i} k(x,x_{i} ) + } w_{0}$$

where $w$ is the weight,$w_{0}$ is the bias, and $k(x,x_{i} )$ is the kernel function.

In the study, a logistic sigmoid function is used as the following formula:

6 $$\sigma \left\{ {g(x_{i} ;w)} \right\} = \frac{1}{{1 + e^{{ - g(x_{i} ;w)}} }}$$

Then, the likelihood of the dataset is expressed as the following formula:

7 $$p(t|w) = \prod\limits_{i = 1}^{N} {\sigma \left\{ {g(x_{i} ;w)} \right\}^{{t_{i} }} \left( {1 - \sigma \left\{ {g(x_{i} ;w)} \right\}} \right)^{{1 - t_{i} }} }$$

The maximum likelihood estimation can be expressed by solving the weight *w* corresponding to the maximization of the following formula:

8 $$\log \left\{ {p(t|w)p(w|a)} \right\} = \sum\limits_{i = 1}^{N} {t_{i} } \log g(x_{i} ;w) - (1 - t_{i} )\log (1 - g(x_{i} ;w)) - \frac{1}{2}w^{T} Aw$$

where $$A$$ = *diag* $$\left( {a_{0} ,a_{1} , \ldots ,a_{n} } \right)$$.

By using Laplace method, the logarithmic posterior probability is approximated twice. Perform two derivations of Eq. (8) to obtain the following formula:

9 $$\nabla_{w} \nabla_{w} \log p(w|t,a)|_{w} = - (\psi^{T} B\psi + A)$$

where $B$ is the diagonal matrix.

An improvement on the fast marginal likelihood maximization method, which can also learn the location and scale parameters of the kernel in the training process. In some real-time applications, it is not only necessary to use a large amount of data for training, but also to update the learning model when the training data arrives. It is very important to introduce incremental learning strategy into the classifier. The original formulation of RVM is extended to design incremental RVM. Incremental learning helps to simplify time calculation and increase algorithm generalization.

In incremental processing in relevance vector machine algorithm, the marginal likelihood can be obtained as the following formula:

10 $$p(t|x,a) = p(t|x,(\Delta^{T} B\Delta + A)^{ - 1} \Delta^{T} Bt)p((\Delta^{T} B\Delta + A)^{ - 1} \Delta^{T} Bt|a)(2\pi )^{{{N \mathord{\left/ {\vphantom {N 2}} \right. \kern-0pt} 2}}} \sqrt {\left| {(\Delta^{T} B\Delta + A)^{ - 1} } \right|}$$

where $\Delta$ is the design matrix.

## Fault detection process of gearbox by multivariate extended variational mode decomposition-based time–frequency images and incremental RVM algorithm

**Figure 3:**
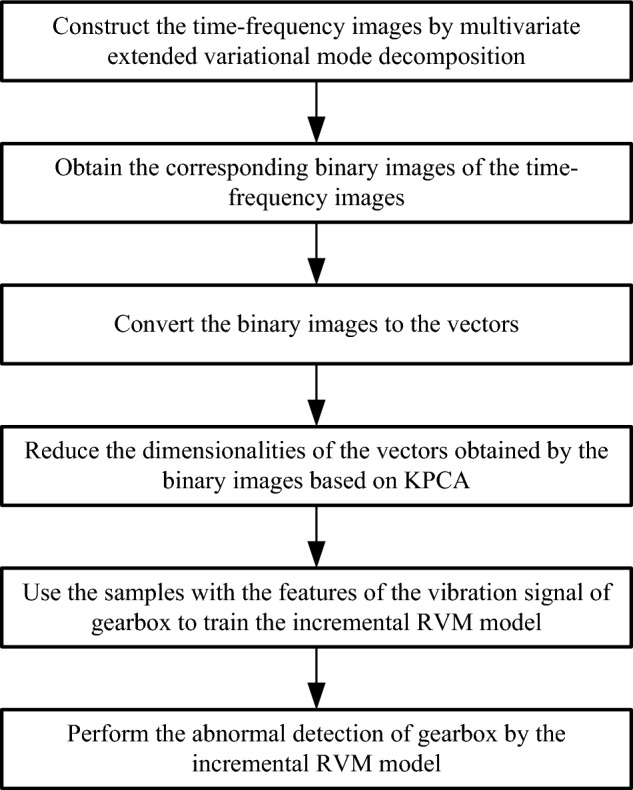
Fault detection process of gearbox by multivariate extended variational mode decomposition-based time–frequency images and incremental RVM algorithm.

Fault detection process of gearbox by multivariate extended variational mode decomposition-based time–frequency images and incremental RVM algorithm is given in Fig. 3. The time–frequency images are constructed by multivariate extended variational mode decomposition. The corresponding binary images of the time–frequency images are obtained, and the binary images are converted to the vectors^20^. The dimension of the vectors obtained by the binary images is very high, which has a great influence on detection results. Thus, the vectors obtained by the binary images need dimensionality reduction. Kernel principal component analysis (KPCA) is used to reduce the dimensionalities of the vectors obtained by the binary images. The essence of kernel function is to simplify the processing of a mapping relationship. It is difficult for us to calculate the function that originally maps the nonlinear separable point set, and it is also very troublesome. The appearance of kernel function is to use the inner product of the point set to simplify this function. On the other hand, KPCA performs the classic PCA cut in the new space after the point set is mapped through kernel function. The vectors after dimension reduction are used as the features of the vibration signal of gearbox. The samples with the features of the vibration signal of gearbox are used to train the incremental RVM model, and the Fault detection of gearbox is performed by the incremental RVM model.

## Experimental testing and results

The vibration signals of gearbox have been measured with acceleration sensor. The vibration signals of gearbox with healthy,abrasion, pitting and snaggletooth are respectively collected under the situation of small load, and the vibration signals of gearbox with healthy,abrasion, pitting and snaggletooth are respectively collected under the situation of big load. 300 training samples including 75 samples denoting healthy, 75 samples denoting abrasion, 75 samples denoting pitting, and 75 samples denoting snaggletooth are used as the training samples, and 200 testing samples including 50 samples denoting healthy, 50 samples denoting abrasion, 50 samples denoting pitting, and 50 samples denoting snaggletooth are used as the testing samples under the situation of small load. Furthermore, the same number of training samples and testing samples are used under the situation of big load. Variational mode decomposition-based time–frequency images and incremental RVM algorithm (VMDTFI–IRVM), Variational mode decomposition and RVM algorithm (VMD–RVM), and traditional RVM algorithm are used to compared with multivariate extended variational mode decomposition-based time–frequency images and incremental RVM algorithm (MEVMDTFI–IRVM).

**Figure 4:**
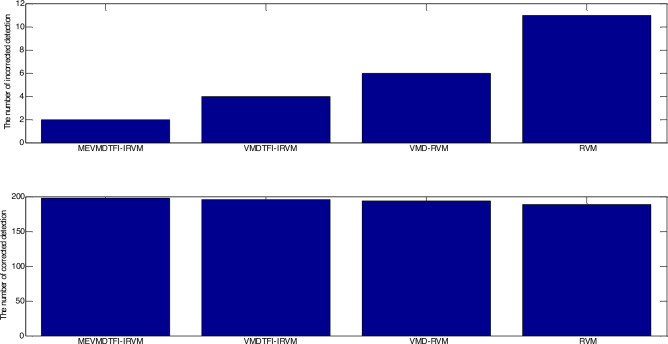
Comparison of detection results among MEVMDTFI–IRVM, VMDTFI–IRVM, VMD–RVM, and RVM under the situation of small load.

**Figure 5:**
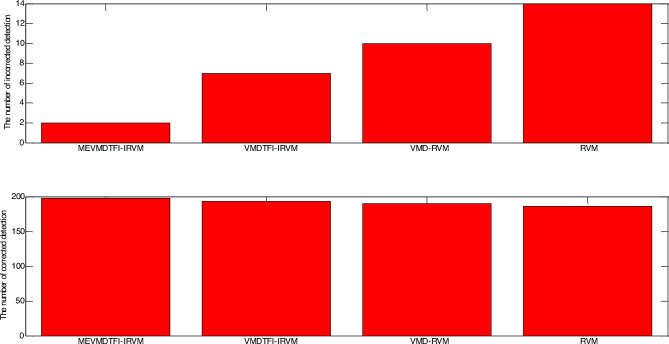
Comparison of detection results among MEVMDTFI–IRVM, VMDTFI–IRVM, VMD–RVM, and RVM under the situation of big load.

As shown in Fig. 4, the number of the testing samples with incorrect detection of MEVMDTFI–IRVM is 2, the number of the testing samples with incorrect detection of VMDTFI–IRVM is 4, the number of the testing samples with incorrect detection of VMD-RVM is 6, and the number of the testing samples with incorrect detection of traditional RVM algorithm is 11 under the situation of small load. As shown in Fig. 5, the number of the testing samples with incorrect detection of MEVMDTFI–IRVM is 2, the number of the testing samples with incorrect detection of VMDTFI–IRVM is 7, the number of the testing samples with incorrect detection of VMD–RVM is 10, and the number of the testing samples with incorrect detection of traditional RVM algorithm is 14 under the situation of big load. It can be seen that the number of the testing samples with incorrect detection of MEVMDTFI–IRVM is less than that of VMDTFI–IRVM, VMD–RVM, and traditional RVM algorithm regardless of small load or big load. Furthermore, as shown in Table 1, the detection accuracy of MEVMDTFI–IRVM is 99%, the number of the testing samples with incorrect detection of VMDTFI–IRVM is 98%, the number of the testing samples with incorrect detection of VMD–RVM is 97%, and the number of the testing samples with incorrect detection of traditional RVM algorithm is 94.5% under the situation of small load. As shown in Table 2, the detection accuracy of MEVMDTFI–IRVM is 99%, the number of the testing samples with incorrect detection of VMDTFI–IRVM is 96.5%, the number of the testing samples with incorrect detection of VMD–RVM is 95%, and the number of the testing samples with incorrect detection of traditional RVM algorithm is 93% under the situation of big load. It can be seen that the detection accuracy of MEVMDTFI–IRVM is less than that of VMDTFI–IRVM, VMD–RVM, and traditional RVM algorithm regardless of small load or big load.The testing results demonstrate that the detection results of MEVMDTFI–IRVM for gearbox are stable, in addition, the detection results of MEVMDTFI–IRVM for gearbox are better than those of VMDTFI–IRVM, VMD–RVM, and traditional RVM algorithm. Furthermore, the testing results demonstrate that multivariate extended variational mode decomposition has better feature extraction ability than traditional variational mode decomposition, and the incremental RVM algorithm is presented for fault detection of gearbox, which is better generalization ability than traditional RVM algorithm.

**Table 1:** Comparison of detection accuracies among MEVMDTFI–IRVM, VMDTFI–IRVM, VMD–RVM, and RVM under the situation of small load.

Method	Accuracy (%)
MEVMDTFI–IRVM	99
VMDTFI–IRVM	98
VMD–RVM	97
RVM	94.5

**Table 2:** Comparison of detection accuracies among MEVMDTFI–IRVM, VMDTFI–IRVM, VMD–RVM, and RVM under the situation of big load.

Method	Accuracy (%)
MEVMDTFI–IRVM	99
VMDTFI–IRVM	96.5
VMD–RVM	95
RVM	93

## Conclusion

This paper presents a novel detection method based on multivariate extended variational mode decomposition-based time–frequency images and incremental RVM algorithm for fault detection of gearbox. The contributions of this paper are shown as follows:

1. The time–frequency images are constructed by multivariate extended variational mode decomposition, compared with single-variable modal decomposition method, multivariate extended variational mode decomposition not only has an accurate mathematical framework, but also has good robustness to non-stationary multi-channel signals with low signal-to-noise ratio.
2. The incremental RVM algorithm is presented for fault detection of gearbox based on the time–frequency images constructed by multivariate extended variational mode decomposition.

The testing results demonstrate that the detection results of MEVMDTFI–IRVM for gearbox are stable, in addition, the detection results of MEVMDTFI–IRVM for gearbox are better than those of VMDTFI–IRVM, VMD–RVM, and traditional RVM algorithm. The detection system for gearbox based on MEVMDTFI–IRVM is developed in the further.

## Data Availability

The datasets used during the current study available from the corresponding author on reasonable request.
